# A Double-Blind Controlled Study to Evaluate the Effects of Yogurt Enriched with *Lactococcus lactis* 11/19-B1 and *Bifidobacterium lactis* on Serum Low-Density Lipoprotein Level and Antigen-Specific Interferon-γ Releasing Ability

**DOI:** 10.3390/nu10111778

**Published:** 2018-11-16

**Authors:** Kyoko Nishiyama, Takahiro Kobayashi, Yuko Sato, Yoshihisa Watanabe, Riki Kikuchi, Ryoko Kanno, Tetsuo Koshizuka, Nozomu Miyazaki, Ken Ishioka, Tatsuo Suzutani

**Affiliations:** 1 Department of Microbiology, Fukushima Medical University School of Medicine, Fukushima 960-1295, Japan; kyoko@fmu.ac.jp (K.N.); tkobayas@fmu.ac.jp (T.K.); yukosato@fmu.ac.jp (Y.S.); ryoko-k@fmu.ac.jp (R.K.); koshizuka-te@gifu-pu.ac.jp (T.K.); m-nozomu@fmu.ac.jp (N.M.); ishiken@fmu.ac.jp (K.I.); 2 Tohoku Kyodo Milk Co., Ltd., Motomiya 969-1104, Japan; watanabe_zen@tk-holstein.com (Y.W.); kikuchi_r@tk-holstein.com (R.K.)

**Keywords:** yogurt, *Lactococcus lactis*, low-density lipoprotein, double-blind controlled study

## Abstract

In order to clarify the effects of the *Lactococcus lactis* (*L. lactis*) 11/19-B1 strain, a double-blind controlled study of yogurt fermented with the strain was carried out. For the study, two kinds of yogurt, the control and test yogurt, were prepared; the control yogurt was fermented with *Streptococcus thermophiles*, *Lactobacillus delbrueckii* subspecies bulgaricus, and *Lactobacillus acidophilus*, and the test yogurt was enriched with *L. lactis* 11/19-B1 and *Bifidobacterium lactis* (*B. lactis*) BB-12 strains. Seventy-six volunteers who had not received treatment with pharmaceuticals were randomly divided into two groups with each group ingesting 80 g of either the test or control yogurt every day for 8 weeks. Before and after yogurt intake, fasting blood was taken and blood sugar, blood lipids, and anti-cytomegalovirus cellular immunity were estimated. In the test yogurt group, low-density lipoprotein (LDL) was significantly decreased (159.1 ± 25.7 to 149.3 ± 24.4; *p* = 0.02), but this effect was not observed in the control yogurt group. When the test yogurt group was divided into two groups based on LDL levels of over or under 120 mg/dL, this effect was only observed in the high LDL group. No LDL-lowering effect of *B. lactis* BB-12 strain was previously reported; therefore, the hypocholesterolemic effects observed in this study are thought to be caused by the *L. lactis* 11/19-B1 strain alone or its combination with the *B. lactis* BB-12 strain.

## 1. Introduction

The processing of milk by fermentation was developed as early as the sixth millennium before centry in order to produce reduced-lactose milk products in lactose-intolerant prehistoric communities and to keep milk in a non-perishable form [1]. In 1905, Elie Metchnikoff suggested a novel function of fermented milk products, particularly yogurt fermented with lactobacilli, in increasing longevity and clarifying the presence of lactobacilli in the colon [2]. Thereafter, many human studies on the disease-preventative and anti-pathogenic activities of yogurt were performed. Recent studies have focused on the function of specific bacterial strains used in fermentation in providing relief from specific diseases or modifying physiological functions as probiotics [3].

*Lactococcus lactis* (*L. lactis*) is a homofermentative species of lactic acid bacteria that produces lactic acid alone from sugars. This species has been widely used in the production of buttermilk, cheese, and other products by reason of its production of various bacteriocins such as nisin, lactococcin, and diacetin [4,5]. Based on this antibacterial activity, *L. lactis* was also investigated as an inhibitor of other bacteria [6]. 

The *L. lactis* 11/19-B1 strain, which was isolated from the surface of kiwi fruit, was estimated to be the strongest immune-stimulator among the 23 lactate-fermenting Gram-positive bacteria tested to date using the silkworm larva muscle contraction assay [7,8]. Further, silkworms ingesting a diet mixed with the 11/19-B1 strain or yogurt fermented with the 11/19-B1 strain showed activation of their innate immunity and enhanced tolerance against *Pseudomonas aeruginosa* infection. These results suggested that this strain could be applied to the production of other fermented foods expected to be probiotic immune-stimulators. 

Yogurt is defined as milk fermented with *Streptococcus thermophiles* (*S. thermophiles*) and any *Lactobacillus* species [9]. In this study, we used a yogurt fermented with *S. thermophiles*, *Lactobacillus delbrueckii* subspecies bulgaricus (*L. bulgaricus*), and *Lactobacillus acidophilus* (*L. acidophilus*) as a control for a comparison of the effects of a novel yogurt enriched with both *L. lactis* 11/19-B1 and *Bifidobacterium lactis* (*B. lactis*) BB-12 strains on immunity and metabolic syndrome in a randomized double-blind controlled trial.

## 2. Materials and Methods

### 2.1. Subjects

The subjects consisted of 47 women (aged between 23 and 66 years) and 29 men (aged between 20 and 59 years) recruited by poster, who had been found to have slightly elevated blood lipid or blood sugar levels by a health examination, but had not received treatment with pharmaceuticals. 

The study was conducted according to the guidelines laid down in the Declaration of Helsinki, and all procedures involving human subjects were approved by the Ethics Committee of Fukushima Medical University. Written consent was obtained from all subjects prior to enrollment in the study. This trial was registered as ID number R000038716 in the University Hospital Medical Information Network (UMIN; http://www.umin.ac.jp/english). 

### 2.2. Study Design

A randomized double-blind controlled trial was designed to compare the effects on blood lipids, blood sugar, and immunological response against human cytomegalovirus (HCMV) of a control yogurt fermented with 3 basic bacteria (*L. bulgaricus*, *S. thermophiles*, and *L. acidophilus*) and to test yogurt fermented with the 3 basic bacteria and two additional bacteria, the *L. lactis* 11/19-B1 strain and the *B. lactis* BB-12 strain. 

At the beginning of the study, fasting serum was analyzed for total cholesterol, high-density lipoprotein (HDL), low-density lipoprotein (LDL), fasting blood sugar, hemoglobin A1c, glyco-albumin, and anti-human cytomegalovirus (HCMV) immunity. After classifying the volunteers into large groups by age and sex, they were divided into two groups based on the LDL values within the groups as there were no major differences in the blood sugar, hemoglobin A1c, and glyco-albumin levels (Table 1). One group ingested 80 g of the test yogurt and the other group ingested 80 g of the control yogurt every day for 8 weeks. The timing of yogurt ingestion during the day was not fixed. During the study period, the volunteers were asked not to consume other probiotic foods or drugs affecting blood lipid and blood sugar levels. At the end of the study period, fasting blood was taken again and analyzed for the same parameters as at the beginning of the study. We started the study with 79 volunteers; however, three of them dropped out without participating in the blood sampling or probiotic foods feeding.

### 2.3. Yogurts

Both the control and the test yogurts were produced once a week by Tohoku Kyodo Milk Industry Co., Ltd. (Fukushima, Japan) and sent to volunteers at 4 °C. There were no differences in the nutritional value, taste, or packaging between the two yogurts. 

### 2.4. Human Cytomegalovirus-Specific Interferon-γ Release Test

The cellular immune status was estimated using the HCMV-specific interferon-γ release test (IFN-γ release test), as over 80% of the adult Japanese population are latently infected with HCMV and their cellular immunity plays a dominant role in HCMV suppression [10,11,12]. The IFN-γ release test was, therefore, applied as an indicator of general cellular immune status in this study. 

**Figure 1:**
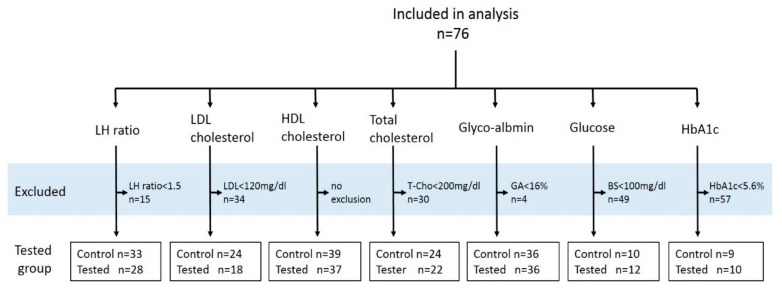
Summary of the subjects. The exclusion criteria are summarized in the gray highlighted section.

Six hundred and seventy ng of pp65 polypeptide (Miltenyi Biotec., Bergisch Gladbach, Germany) in 10 μL of Roswell Park Memorial Institute (RPMI) 1640 medium was added to 100 μL samples of heparinized peripheral venous blood in a 96-well plate. After incubation for 24 h at 37 °C in a CO_2_ incubator, the cultures were centrifuged at 800× *g* at 4 °C for 10 min with a micro-centrifuge, and the supernatants were collected and stored at −80 °C until IFN-γ measurement. The amount of IFN-γ in the supernatant was quantified by ELISA assay using Human IFN-γ ELISA Ready-SET-GO!^®^ (eBioscience, San Diego, CA, USA) in accordance with the manufacturer’s protocol. Subjects secreting over 600 ng/mL of IFN-γ in the assay before the study period were excluded from the analysis as their immunity had been stimulated by factors such as a common cold (*n* = 16, Figure 1). One person, whose IFN-γ titer was over 10,000 ng/dL after yogurt intake, was also excluded from the analysis. 

### 2.5. Statistical Analysis

All data are presented as a mean ± SD. Statistically significant differences were analyzed by two-way ANOVA using SPSS ver. 24 software (IBM, Armonk, NY, USA). Statistical significance was set at *p* ≤ 0.05 on two-tailed analyses.

## 3. Results

### 3.1. Effect of Yogurt Intake on Serum Parameters

Subject characteristics at the beginning of the study are summarized in Table 1.

Intake of the control yogurt had no significant effect on blood lipid or sugar levels except for a slight elevation in HbA1c (Table 2). On the other hand, ingestion of the test yogurt led to a significant decrease in LDL cholesterol by approximately 10 mg/dL, leading to a decrease in the total cholesterol value and LDL/HDL ratio.

Moreover, the effect of yogurt was analyzed in selected volunteers whose serum parameters were slightly higher than the normal level. The criteria for selection of these volunteers were total cholesterol ≥ 200 mg/dL, LDL ≥ 120 mg/dL, LDL/HDL ≥ 1.5, glucose ≥ 100 mg/dL, glyco-albumin ≥ 16%, or hemoglobin A1_C_ ≥ 5.6% (Figure 1). 

Each value was compared between the control and test groups by two-way ANOVA to exclude the effects of sex, age, and other factors (Table 3). 

Among the tested items, LDL was significantly decreased, which consequently lowered the total cholesterol value and LDL/HDL ratio, and glyco-albumin was elevated in the test group, although no effects were observed in the control group. In contrast, the test yogurt did not show any effect on LDL in the LDL cholesterol normal group (Table 4).

### 3.2. Effect of Yogurt Intake on Cellular Immunity

**Figure 2:**
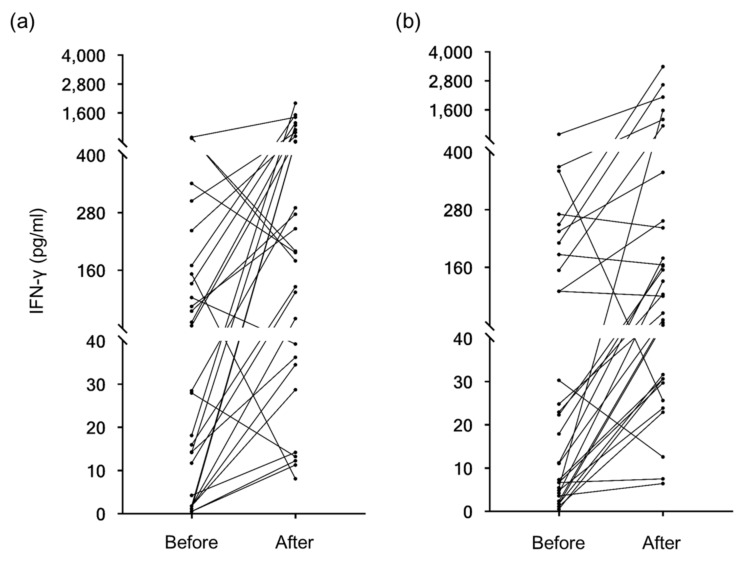
Results of the IFN-γ release test before and after yogurt ingestion for 8 weeks. Changes in the amount of IFN-secretion from T cells by stimulation with human cytomegalovirus (HCMV)-specific antigen are shown. (**a**) Before and after control yogurt or (**b**) 11/19-B1 yogurt ingestion.

The effect of yogurt ingestion on cellular immunity was estimated using the IFN-γ release test. Variable immunological responses were observed in individuals with yogurt intake as the standard deviations were very large (Figure 2). However, both yogurts significantly enhanced host cellular immunity, with a *p*-value of 0.037 for the control yogurt and 0.029 for the test yogurt according to two-way ANOVA in the groups after exclusion of the volunteers with extremely high IFN-γ (Table 3). 

## 4. Discussion

After the publication of Metchnikoff’s innovative theory, various functions of yogurt related to the maintenance of health have been studied including the prevention of certain kinds of cancer [12] and infectious diseases, such as *Helicobacter pylori* infection [13,14] and viral infections [15,16], decline in serum lipids [17,18], anti-obese effect [19], and improvement in the symptoms of allergic disease [20]. In these studies, many kinds of lactic acid bacteria species and strains were studied, and different functions were observed even in the same species. In this study, we studied the effects of a combination of two species, a novel *L. lactis* strain 11/19-B1 and *B. lactis* BB-12 strain, with a special focus on the prevention of metabolic syndrome and enhancement of cellular immunity.

The *B. lactis* BB-12 stain originates from Chr. Hansen’s collection of dairy cultures and is one of most studied probiotic bacterial strains. Over 300 papers have been published on this strain’s potential to inhibit pathogens, enhance intestinal barrier function, improve bowel function, and stimulate immunity [15]. Based on these functions, it was proved that the BB-12 strain can prevent diarrhea and constipation and shorten the duration of infections [21,22]. On the other hand, neither single nor combined supplementation of the BB-12 strain with the *L. acidophilus* La5 strain showed any hypocholesterolemic effects in two clinical randomized control studies [23,24]. Therefore, the hypocholesterolemic effects observed in this study are thought to be caused by the *L. lactis* 11/19-B1 strain alone or its combination with the *B. lactis* BB-12 strain. The most important aspect of this effect is that it was observed in the hypocholesterolemic volunteers, but not in those with normal levels of serum cholesterol. Anderson and Gilliland hypothesized that every 1% reduction in serum cholesterol concentration is associated with an estimated 2 to 3% reduction in the risk of coronary heart disease [25]. The regular intake of fermented milk containing an appropriate strain of *L. acidophilus* has the potential to reduce the risk of coronary heart disease by 6 to 10%, with an additional effect expected for the consumption of 11/19-B1 yogurt.

Previous studies found that *L. lactis*, even heat-killed bacteria, delayed aging through modification of intestinal flora [26,27]. Further study is required to determine whether the hypocholesterolemic effect observed in this study is also related with the modification of intestinal flora or if it is a direct function of the strain and whether the function is unique to the 11/19-B1 strain or a common feature of *L. lactis*. 

The results of this study demonstrate that the 11/19-B1 yogurt acts as an immune-enhancer. To date, many previous studies reported that various lactic acid bacterial species and strains possessed immune-enhancing activity; for example, the enhancement of IgA production by Bifidobacteria [28,29], natural killer cell activity by *Lactobacillus casei* Shirota strain [30], and Th1 and Treg by *Lactobacillus pentosus* strain S-PT84 [31]. Though we could not demonstrate that the 11/19-B1 yogurt enhanced immunity in comparison with the control, some unique functions, such as the activation of dendritic cells in *L. lactis* JCM5805 strain, were reported previously [32]. Therefore, further study of the 11/19-B1 strain is ongoing. 

## 5. Conclusions

The results of this study demonstrate that the enrichment of yogurt with both *L. lactis* 11/19-B1 and *Bifidobacterium lactis* BB-12 strains can act to lower serum LDL cholesterol and enhance the function of the control yogurt as a stimulator of cellular immunity. These results indicate that the combination of *L. lactis* 11/19-B1 and *B. lactis* strains affords a useful candidate as a starter for daily probiotics. 

## Figures and Tables

**Table 1:** Subject characteristics.

	Control Yogurt Group (*n* = 39)	Test Yogurt Group (*n* = 37)	*p* Value
Age	43.8 ± 11.7	40.9 ± 10.6	0.27 *
Sex (% of male)	38.5	37.8	0.96 **
Total cholesterol (mg/dL)	214.3 ± 36.5	215.3 ± 34.2	0.90 *
LDL cholesterol (mg/dL)	129.7 ± 35.0	133.0 ± 31.3	0.67 *
HDL cholesterol (mg/dL)	61.9 ± 14.0	63.6 ± 16.4	0.67 *
LDL/HDL ratio	1.97 (1.16–4.83)	2.21 ± 0.92	0.65 ***
Glucose (mg/dL)	94.5 (78–133)	95.0 (80–140)	0.85 ***
Glyco-albumin (%)	13.9 ± 1.4	13.7 ± 1.1	0.43 ***
HbA1c (%)	5.4 ± 0.3	5.3 (4.9–6.8)	0.81 ***
IFN-γ (pg/mL)	84.6 (0.5–8702.5)	30.3 (0.5–5696.4)	0.59 ***

* Two-way ANOVA; ** χ^2^ test; *** Mann-Whitney U test.

**Table 2:** Effect of yogurt on serum parameters.

	Control Yogurt Group (*n* = 39)	*p* Value *	Test Yogurt Group (*n* = 37)	*p* Value *
Total cholesterol (mg/dL)	209.8 ± 34.8	0.06	198.0 (150–303)	<0.001
LDL cholesterol (mg/dL)	130.5 ± 31.3	0.26	123.2 ± 31.9	0.01
HDL cholesterol (mg/dL)	61.9 ± 14.4	0.79	62.7 ± 14.5	0.54
LDL/HDL ratio	2.21 ± 0.70	0.09	1.92 (0.90–4.34)	0.02
Glucose (mg/dL)	94.0 (80–199)	0.19	95.0 (68–140)	0.17
Glyco-albumin (%)	13.7 ± 1.4	0.16	13.8 ± 1.0	0.85
HbA1c (%)	5.5 (4.9–6.2)	0.01	5.4 (5.0–6.6)	0.32
IFN-γ	276.9 (8.1–10255.7)	0.42	154.9 (6.5–4592.5)	0.46

* Two-way ANOVA.

**Table 3:** Effect of yogurt in volunteers showing slightly elevated values for each parameter.

	Group	Before	After	*p* Value *
Total cholesterol (mg/dL)	Control (*n* = 24)	236.2 ± 24.9	228.0 ± 29.0	0.04
	Test (*n* = 22)	236.7 ± 26.5	225.1 ± 30.6	<0.001
LDL ** cholesterol (mg/dL)	Control (*n* = 24)	151.8 ± 23.4	146.8 ± 24.7	0.14
	Test (*n* = 18)	159.1 ± 25.7	149.3 ± 24.4	0.02
HDL ** cholesterol (mg/dL)	Control (*n* = 39)	61.9 ± 14.0	61.8 ± 14.4	0.92
	Test (*n* = 37)	63.6 ± 16.3	62.7 ± 14.5	0.52
LDL/HDL ratio	Control (*n* = 33)	2.5 ± 0.8	2.4 ± 0.6	0.12
	Test (*n* = 28)	2.5 ± 0.8	2.4 ± 0.8	<0.001
Glucose (mg/dL)	Control (*n* = 10)	112.4 ± 9.8	108.0 ± 9.8	0.17
	Test (*n* = 12)	108.1 ± 11.6	106.4 ± 12.7	0.25
Glyco-albumin (%)	Control (*n* = 36)	13.7 ± 1.2	13.7 ± 1.1	0.39
	Test (*n* = 36)	13.6 ± 0.9	13.8 ± 1.0	0.03
HbA1c (%)	Control (*n* = 9)	5.7 ± 0.2	5.7 ± 0.3	0.83
	Test (*n* = 10)	5.9 ± 0.4	5.8 ± 0.5	0.14
IFN-γ **	Control (*n* = 29)	121.3 ± 177.5	765.6 ± 1837.9	0.037
	Test (*n* = 30)	101.2 ± 144.8	472.1 ± 847.6	0.029

* Two-way ANOVA. ** LDL, low-density lipoprotein; HDL, high-density lipoprotein; IFN-γ, interferon-γ.

**Table 4:** Effect of yogurt on the LDL values of the LDL normal (<120 mg/dL) volunteers.

Group	Number	Before	After	*p* Value *
Control yogurt	15	102.9 ± 13.3	104.4 ± 21.6	0.93
Test yogurt	19	101.8 ± 11.7	98.6 ± 21.5	0.53

* Two-way ANOVA.
